# A review of the therapeutic role of the new third-generation TKI olverembatinib in chronic myeloid leukemia

**DOI:** 10.3389/fonc.2022.1036437

**Published:** 2022-12-08

**Authors:** Honglan Qian, Dongxu Gang, Xiaoyu He, Songfu Jiang

**Affiliations:** Department of Hematology, The First Affiliated Hospital of Wenzhou Medical University, Wenzhou, Zhejiang, China

**Keywords:** olverembatinib, chronic myeloid leukemia, tyrosine kinase inhibitors, breakpoint cluster region protein and abelson tyrosine-protein kinase 1, T315I mutation

## Abstract

Several tyrosine kinase inhibitors (TKIs) have been developed as targeted therapies to inhibit the oncogenic activity of several tyrosine kinases in chronic myeloid leukemia (CML), acute lymphoid leukemia (ALL), gastrointestinal stromal tumor (GIST), and other diseases. TKIs have significantly improved the overall survival of these patients and changed the treatment strategy in the clinic. However, approximately 50% of patients develop resistance or intolerance to imatinib. For second-generation TKIs, approximately 30%–40% of patients need to change therapy by 5 years when they are used as first-line treatment. Clinical study analysis showed that the T315I mutation is highly associated with TKI resistance. Developing new drugs that target the T315I mutation will address the dilemma of treatment failure. Olverembatinib, as a third-generation TKI designed for the T315I mutation, is being researched in China. Preliminary clinical data show the safety and efficacy in treating CML patients harboring the T315I mutation or who are resistant to first- or second-line TKI treatment. Herein, we review the characteristics and clinical trials of olverembatinib. We also discuss its role in the management of CML patients.

## Introduction

Chronic myeloid leukemia (CML) is a clonal hematopoietic stem cell disorder that accounts for approximately 15% of adult leukemia (1). The incidence of CML varies from 0.4/100,000 to 1.75/100,000 persons per year (2). Diagnosis is usually suspected from the complete blood count (CBC) and blood smear. Fluorescence *in situ* hybridization (FISH) for abnormal chromosome t(9;22)(q34;q11.2) and reverse transcriptase quantitative PCR for the BCR-ABL1 fusion gene help in the final diagnosis (3). CML is clinically staged into the chronic phase (CP), accelerated phase (AP), and blast phase (BP). In untreated patients, progression to BP occurs at a median of 3–5 years after initial diagnosis (4).

When a patient is diagnosed with CML, the first-line treatment is a TKI. In an open-label, multicenter trial with a crossover design for patients treated with the first-generation TKI imatinib, the estimated overall survival rate at 10 years was 83.3%, and the cumulative rate of major cytogenetic response (MCyR) at the end of the trial was 89.0%. A total of 82.8% of patients had a complete cytogenetic response (CCyR) (5). Despite the excellent results obtained in clinical trials, approximately 40%–45% of patients discontinue imatinib for various reasons, including unsatisfactory therapeutic outcomes in 16% of patients or primary resistance (6). Dose escalation after failure of imatinib treatment is an important option, though it is likely to be effective only in some subsets of patients. However, it is not recommended in the clinic. For such patients, an important salvage treatment strategy is represented by second-generation TKIs including dasatinib, nilotinib, and bosutinib, which allow recovering a CCyR in about 50% of cases (7–11, **Table 1**). When used as a front-line treatment, second-generation TKIs provide a faster achievement of a higher rate of cytogenetic and molecular responses with respect to imatinib (12–18, 22–24, **Table 1**). Subsequently, second-generation TKIs have been authorized for the first-line treatment of newly diagnosed Philadelphia chromosome–positive (Ph+) adult CP-CML (25). Some patients experience treatment failure with second-generation TKIs and require a switch to a different TKI. Intolerance and resistance are major causes of treatment failure. Resistance to TKIs can arise from BCR-ABL1-dependent mechanisms, such as mutations in the kinase domain, overexpression, or amplification of BCR-ABL1, or BCR-ABL1-independent mechanisms (26). The well-studied and most common mechanism is point mutation involving the BCR-ABL1 kinase domain (27). In one study, which included 175 patients with newly diagnosed CML or resistance to TKIs, 28 different mutations were detected in 54 (30.86%). A total of 14 (8.0%) patients carried the T315I mutation, accounting for the largest proportion in the mutation group (28). It has been demonstrated that the T315I mutation is insensitive to all first- and second-generation TKIs. Asciminib and ponatinib (19–21, **Table 1**), as new-generation TKIs, have unique activity against the T315I mutation and are approved to treat patients with resistance or intolerance to prior TKI therapy or the presence of the BCR-ABL1 T315I mutation in all CML phases. Research has shown that the ATP-binding site mutation T315M confers resistance to ponatinib (29). *In vitro* data indicated that some compound mutations involving T315I also potentially impact ponatinib sensitivity (30). New TKIs have higher sensitivity to the T315I mutation. In November 2021, olverembatinib, as a new third-generation TKI that showed clinical efficacy in CML patients with the T315I mutation, received its first approval in China for the treatment of adult patients with TKI-resistant CP-CML or AP-CML harboring the T315I mutation (31). It is the first and only approved TKI designed for the T315I mutation in China. Here, we review the characteristics and clinical studies of olverembatinib and its application value in the clinic.

**Table 1:** TKI treatment of CP-CML patients.

Trial	Patient number (N)	Treatment	Efficacy	Reference
**START-C**	387 CP-CML Imatinib failure	Dasatinib 70 mg BID	CHR 90% CCyR 53% MMR 47%	7
**START-R** **NCT00103844**	150 CP-CML Imatinib failure	Dasatinib 70 mg BID	CHR 93% MCyR 53% CCyR 44%	8
**CA180034** **NCT00123474**	622 CP-CML Imatinib failure	Dasatinib	100 mg QD CHR 92%, CCyR 50% 140 mg QD CHR 87%, CCyR 50% 70 mg BID CHR 88%, CCyR 53% 50 mg BID CHR 92%, CCyR 49%	9
**NCT00109707**	321 CP-CML Imatinib failure	Nilotinib 400 mg BID	CCyR 46% MMR 28%	10
**NCT00261846**	288 CP-CML Imatinib failure	Bosutinib 500 mg QD	CCyR 48% MMR 35%	11
**DASISION** **NCT00481247**	259 CP-CML 260 CP-CML	Dasatinib 100 mg QD Imatinib 400 mg QD	CCyR 86%, MMR 64% CCyR 82%, MMR 46%	12
**DASCERN** **NCT01593254**	174 CP-CML Suboptimal response to imatinib	Dasatinib 100 mg QD	MMR 64%	13
**ENESTnd** **NCT00471497**	846 CP-CML	Nilotinib Imatinib	Nilotinib 300 mg BID CCyR 80% Nilotinib 400 mg BID CCyR 78% Imatinib 400 mg QD CCyR 64%	14
**MDACC**	93 CP-CML	Dasatinib 100 mg QD 50 mg BID	CHR 98% CCyR 95% MMR 87%	15
**S0325** **NCT00070499**	246 CP-CML	Dasatinib 100 mg Imatinib 400 mg	CHR 81%, CCyR 84%, MMR 59% CHR 82%, CCyR 69%, MMR 44%	16
**BFORE** **NCT02130557**	536 CP-CML	Bosutinib 400 mg QD Imatinib 400 mg QD	MMR 73.9%, CCyR 83.3% MMR 64.6%, CCyR 76.8%	17
**BYOND trial** **NCT02228382**	163 CP-CML Resistant or intolerant to prior TKIs	Bosutinib 500 mg QD	CCyR 80.6%, MMR 70.5%	18
**PACE** **NCT01207440**	267 CP-CML Dasatinib or nilotinib failure	Ponatinib 45 mg QD	MCyR 56%, MMR 34%, CCyR 46%	19
**OPTIC** **NCT02467270**	282 CP-CML Resistant to ≥1 prior TKI therapy	Ponatinib 45 mg QD 30 mg QD 15 mg QD	MCyR 50.5%, MMR 34.4% MCyR 33.3%, MMR 24.7% MCyR 43.8%, MMR 23.1%	20
**ASCEMBL** **NCT03106779**	233 CP-CML Resistant to ≥2 prior TKI therapy	Asciminib 40 mg BID Bosutinib 500 mg QD	MMR 25.5% MMR 13.2%	21

MMR, major molecular response; CHR, complete hematological response; MCyR, major cytogenetic response; CCyR, complete cytogenetic response.

## Characteristics and inhibitory activity of olverembatinib

Olverembatinib tightly binds to the ATP-binding sites of native BCR-ABL1 and multiple BCR-ABL1 mutants, including Q252H, E255K, F317L, F317I M351T, H396P, and the most refractory gatekeeper mutant T315I (32). It binds to phosphorylated and non-phosphorylated forms of BCR-ABL1 kinase, as well as to several other kinases, including KIT, FLT3, fibroblast growth factor receptor 1 (FGFR1), and platelet-derived growth factor receptor α (PDGFRα). In the predicted binding pose of olverembatinib (GZD824) with activated BCR-ABL1^T315I^, the 1H-pyrazolo[3,4-b] pyridine core occupies the adenine pocket of BCR-ABL1 kinase to form a hydrogen bond donor-acceptor network. The alkyne moiety of GZD824 presents favorable van der Waals interactions with BCR-ABL T315I (32).

*In vitro*, olverembatinib strongly inhibits the proliferation of Ba/F3 cells expressing BCR-ABL1^T315I^, as well as K562/Ku812 CML cells and SUP-B15 ALL cells expressing BCR-ABL1. In mouse allograft leukemia models, it inhibited tumor growth in the allograft model using Ba/F3 cells expressing BCR-ABL1^WT^ or BCR-ABL1^T315I^ in a dose-dependent manner and showed overall survival benefits (33). Moreover, in FLT3-ITD mutant AML and xenograft tumor models, cell growth was significantly inhibited with olverembatinib (34, 35). Olverembatinib also exhibits antileukemic activity in imatinib-resistant/sensitive (GIST) cell lines and a GIST mouse model by inhibiting the phosphorylation of KIT and its downstream proteins, including AKT, ERK1/2, and STAT3 (36). The above studies demonstrate that olverembatinib has wide antitumor activities and exerts a strong inhibitory effect.

## Clinical trials of olverembatinib

### CP-CML

In an open-label, multicenter phase 1/2 trial, 127 Chinese patients with TKI-resistant CP-CML were enrolled (37). The characteristics of the patients are depicted in **Table 2**. Forty-one (43.6%) patients had a single T315I mutation, and 15 (16.0%) patients carried T315I and additional mutations. A total of 20.2% of patients had no BCR-ABL1 mutation. Compound mutations were detected in 7 (7.4%) patients. The median follow-up was 37 months. In evaluable patients without baseline responses, MCyR and CCyR were achieved in 79.3% and 69.4% of patients, respectively, at a median of 3 months. The cumulative 3-year incidences of MCyR, CCyR, MMR, MR4.0, and MR4.5 were 78.6%, 69.0%, 55.9%, 43.5%, and 38.6%, respectively. The probabilities of sustained MCyR, CCyR, and MMR at 3 years were 77.3%, 72.2%, and 76.0%, respectively. Seven patients died of disease progression, other diseases, or unknown reasons. The probabilities of PFS and OS at 3 years were 92% and 94%, respectively.

**Table 2:** Characteristics of patients in the olverembatinib trial (37).

		CP-CML (N = 127)	AP-CML (N = 38)
**Previous treatment**	Imatinib Imatinib/dasatinib Imatinib/nilotinib Imatinib/dasatinib/nilotinib Nilotinib Dasatinib Dasatinib/nilotinib	16 (12.6%) 47 (37%) 22 (17.3%) 35 (27.6%) 4 (3.1%) 1 (0.8%) 2 (1.6%)	6 (15.8%) 13 (34.2%) 4 (10.5%) 10 (26.3%) 2 (5.3%) 1 (2.6%) 2 (5.3%)
**Mutation**	No mutation T315I single mutation T315I and additional mutation Other mutations Compound mutations	19 (20.2%) 41 (43.6%) 15 (16.0%) 12 (12.8%) 7 (7.4%)	1 (4.2%) 12 (50%) 4 (16.7%) 2 (8.3%) 5 (20.8%)
**Ongoing**		96 (75.6%)	18 (47.4%)
**Dose**	20 mg 30 mg 40 mg 50 mg	8 (8.3%) 38 (39.6%) 41 (42.7%) 9 (9.4%)	3 (16.7%) 6 (33.3%) 7 (38.9%) 2 (11.1%)
**Discontinuation**		31 (24.4%)	20 (52.6%)
**Disease progression**		5 (3.9%)	10 (26.3%)
**Treatment failure**		8 (6.3%)	4 (10.5%)
**Death**		1 (0.8%)	2 (5.3%)

### AP-CML

A phase 1/2 study (37) included 38 patients with TKI-resistant BCR-ABL1^T315I^ AP-CML, 12 patients with a single T315I mutation, and four with T315I and additional mutations. Among 37 patients without baseline MaHR, 27 patients experienced CHR at a median of 3 months. The three-year cumulative incidences of achieving MCyR, CCyR, MMR, MR4.0, and MR4.5 were 47.4%, 47.4%, 44.7%, 39.3%, and 32.2%, respectively. The probabilities of PFS and OS at three years were 60% and 71%, respectively. A total of 11 patients had CML that progressed to the blast phase, and 2 patients died. We also summarize some characteristics in **Table 2**. These studies show that olverembatinib is as efficacious and well tolerated as monotherapy in patients with TKI-resistant BCR-ABL1^T315I^ AP-CML.

### Ongoing clinical trials in CML

In the phase I dose escalation study (SJ-0002), patients with TKI-resistant CML were enrolled in 11 dose escalation cohorts ranging from 1 mg to 60 mg every other day. The maximum tolerated dose (MTD) has been established as 50 mg (38). Olverembatinib is also being assessed in patients with CP, AP, and BP-CML or with Ph+ ALL who have experienced resistance or intolerance to at least two TKIs or in subjects with Ph+ BCP ALL or LBP CML who have experienced resistance or intolerance to at least one second- or later-generation TKI (NCT04260022). In a phase 2 study, the efficacy and safety of Olverembatinib in CP-CML patients who are resistant or intolerant to first- and second-generation TKI are being evaluated (NCT04126681). In this study, the available treatment including hydroxyurea or interferon-based therapy, homoharringtonine, imatinib, dasatinib, or nilotinib will be selected by the investigator for each participant. The efficacy of olverembatinib will be determined by evaluating the patients’ event-free survival (EFS). In addition, in one phase III study (NCT05311943), 40 patients are recruited to evaluate the efficacy and safety of olverembatinib in patients with CP-CML who are resistant or intolerant to at least two second-generation TKIs. The molecular response (MR), MMR, OS, PFS, and adverse events will be measured. We summarize these trials in **Table 3**.

**Table 3:** Clinical studies of olverembatinib.

Registration Number	Types	Phase	Status	Patient Number	Intervention	Main indicators
NCT04260022	CP/AP/BP-CML and Ph+ ALL, Resistance or intolerance to at least two TKIs, BCP-ALL and LBP- CML, Resistance or intolerant to at least one second or later generation TKI	I	Recruiting	62	Blinatumomab Olverembatinib. 30, 40, 50 mg QOD	Phase 2 dose and PK/PD
NCT05376852	AP-CML and CML in lymphoid/myeloid blast crisis	II	Recruiting	40	Olverembatinib,40 mg QOD Combination with decitabine	Overall response rate (end of cycle 2)
NCT05311943	CP-CML, Resistance or intolerance to at least two second generation TKIs	III	Recruiting	40	Olverembatinib, 40 mg QOD	MMR at 12 months
NCT03883087	CP-CML with T315I mutation	II	Active, Not recruiting	41	Olverembatinib,40 mg QOD	MCyR (by the end of cycle 24)
NCT03883100	AP-CML with T315I mutation	II	Active, Not recruiting	23	Olverembatinib,40 mg QOD	MaHR (by the end of cycle 24)
NCT04126681	CP-CML, Resistance or intolerance to first and second generation TKIs	II	Active, Not recruiting	144	Olverembatinib. Hydroxyurea or Interferon-based therapy. Homoharringtonine. Imatinib, Dasatinib or Nilotinib	EFS (by the end of cycle 24)

EFS, event-free survival; PK/PD, pharmacokinetic or pharmacodynamic; BCP-ALL, Ph+ B-cell precursor (BCP) ALL; LBP, lymphoid blast phase CML (CML LBP); AUC, area under the curve; MaHR, major hematologic response. Each cycle is 28 days.

## Adverse effects in patients treated with olverembatinib

In CP-CML with olverembatinib treatment, the treatment-related adverse events were mainly grade 1 or 2, and the most frequent nonhematologic adverse event was skin hyperpigmentation (85%). Grade 3 or 4 nonhematologic adverse events included hypertriglyceridemia (8.7%), increased creatine phosphokinase (7.9%), and hypertension (5.5%). The most common hematologic treatment-related adverse event was thrombocytopenia (73.2%, including 48.8% of patients with grade 3 or 4). A total of 16.5% of patients had grade 3 or higher leukopenia, while 20.5% of patients had anemia (grade 3 or higher). In AP-CML, the common nonhematologic adverse events included skin pigmentation (81.6%), hypocalcemia (60.5%), proteinuria (50%), hyperuricemia (50%), hypertriglyceridemia (34.2%), and hyperphosphatemia (28.9%), of which most were grade 1 or 2. Thrombocytopenia, anemia, leukopenia, and neutropenia were the common hematological adverse events, with incidences of 86.8%, 63.2%, 50%, and 21.1%, respectively; these adverse events were manageable (37).

## Role of olverembatinib in CML

The goals of treatment in CML are the prolongation of survival and prevention of progression toward AP and BP. Over time, the goals of treatment have evolved to achieve deep molecular responses. The focus for patients living with CML has shifted toward improving quality of life and saving costs, and the goal of achieving treatment-free remission is becoming more desirable. Some patients show resistance to first-generation TKIs and must switch to second-generation TKIs. Prospective studies report the superiority of second-generation TKIs, particularly in terms of MR rate, speed, and depth (17, 39–41). Actually, four TKIs, namely, imatinib, nilotinib, dasatinib, and bosutinib, are recommended for first-line treatment.

In patients with TKI treatment-naïve BCR-ABL AP/BP-CML, the standard treatment is the abovementioned four TKIs. In the case of intolerance or failure to one TKI, the choice of the subsequent therapy should also consider whether the patients develop ABL1 point mutations (42). The second-line treatment is a switch to any of the other TKIs. If the patients are resistant or intolerant to one of the second-generation TKIs, in such situations, ponatinib is also recommended. If patients are heavily pretreated and resistant to at least two second-generation TKIs, asciminib as the third-line treatment is an important option (43). If disease progresses to AP or BP during TKI treatment, we recommend choosing third-generation TKIs, bone marrow transplantation or experimental treatment (44). In CML patients with the BCR-ABL1 T315I mutation, the third-generation TKIs ponatinib and asciminib are preferentially recommended (45). Nevertheless, there is no consensus about the choice of treatment strategy. According to our previous studies, olverembatinib shows safety and efficacy in CP and BP-CML patients with at least two generations of TKI resistance or T315I mutations. We further recommend olverembatinib as the third-line treatment (37). In one study, the third-generation TKI ponatinib combined with fludarabine, cytarabine, granulocyte colony-stimulating factor, and idarubicin (FLAG-IDA) was used in Ph+ BP-CML patients, with 69% of patients in the second chronic phase after one cycle of treatment (46). Further studies are needed to investigate the efficacy and safety of olverembatinib in combination with other chemotherapies in AP-CML and BCR-ABL1T315I mutation patients.

## Conclusion

Resistance to TKIs is a complex and multifactorial process that presents the selection of leukemia clones with the ability to evade treatment (47). Some specific mutations result in resistance to one particular TKI. Nilotinib is resistant to Y253H/F, E255K/V, F359V/C/I, and T315I mutations, whereas bosutinib works for all identified mutations with exceptions of V299L and T315I mutations (48, 49). The most common resistance mechanism is the ABL1 kinase domain point mutation. T315I mutation is the most aggressive point mutation identified in BCR-ABL1. Ponatinib was designed to overcome this mutation, while compound mutations, including T315I, cause resistance to ponatinib. Overall, olverembatinib shows a significant inhibitory effect in the presence of the T315I mutation or compound mutations because it does not form a hydrogen bond with the hydroxyl group at this residue (36); it has been demonstrated to be safe and efficacious in phase I/II clinical trials in CP-CML patients resistant to at least two TKIs. In China, olverembatinib is the only third-generation TKI approved for the treatment of CML patients with T315I mutation. Therefore, it is preferentially recommended in CML patients harboring the T315I mutation in China. Besides, olverembatinib as a third-line therapy might represent a viable option for patients failing first- or second-generation TKIs.

## Author contributions

All authors listed have made a substantial, direct, and intellectual contribution to the work and approved it for publication.
